# How I do it: decompressive hemicraniectomy supplemented with resection of the temporal pole and tentoriotomy for malignant ischemic infarction in the territory supplied by the middle cerebral artery

**DOI:** 10.1007/s00701-022-05152-7

**Published:** 2022-02-16

**Authors:** Salah M. M. Sehweil, Zoya Alexandrovna Goncharova

**Affiliations:** Ministry of Health of the Russian Federation, Rostov State Medical University (a Federal Public Higher Education Establishment), 29 Nachichevansky Lane, Rostov-on-Don, 344022 Russia

**Keywords:** Malignant ischemic infarction, Middle cerebral artery, Decompressive hemicraniectomy, Cerebral edema, Cerebellar tentorium

## Abstract

**Abstract:**

Malignant ischemic infarction in the territory supplied by the middle cerebral artery is an extremely severe form of ischemic stroke associated with development of massive uncontrollable postischemic edema of the affected cerebral hemisphere; the end result of which is development of transtentorial herniation and death.

**Method:**

The surgical technique of performance of decompressive hemicraniectomy involves removal of an extensive bone flap in the fronto-temporo-parieto-occipital zone with resection of the temporal squama and of the greater wing of the sphenoid bone to visualize the level of entrance of the middle meningeal artery to the cranial cavity, which, in its turn, allows resection of the upright margin of the middle cranial fossa. Decompressive hemicraniectomy is supplemented with resection of the temporal pole and tentoriotomy.

**Conclusion:**

Performance of decompressive hemicraniectomy in combination with resection of the resection of the temporal pole and tentoriotomy is an effective surgical method of treatment of malignant ischemic stroke in the territory supplied by the middle cerebral artery, capable of reducing the lethality rate during the postoperative period.

**Supplementary Information:**

The online version contains supplementary material available at 10.1007/s00701-022-05152-7.

## Relevant surgical anatomy

Decompressive hemicraniectomy is the most effective method of surgical treatment of malignant ischemic stroke in the territory of the middle cerebral artery (MCA) [2–4, 7]. Decompressive hemicraniectomy consists in resection of an extensive bone flap with a size of least 12 × 12 cm on the side of the affected brain hemisphere, in order to create additional space for the massive postischemic swelling of the brain hemisphere [1]. However, in cases of malignant ischemic infarction in the MCA territory, conventional decompressive hemicraniectomy does not prevent a severe course of the disease and is associated with a high lethality rate [5, 6]. The main cause of mortality in such cases is development of the brain herniation, in particular, transtentorial herniation. The middle meningeal artery is an important surgical landmark for performance of decompressive hemicraniectomy. Resection of the temporal pole and tentoriotomy prevents the development or remediates the effects of transtentorial herniation.

## Description of the technique

**Fig. 1:**
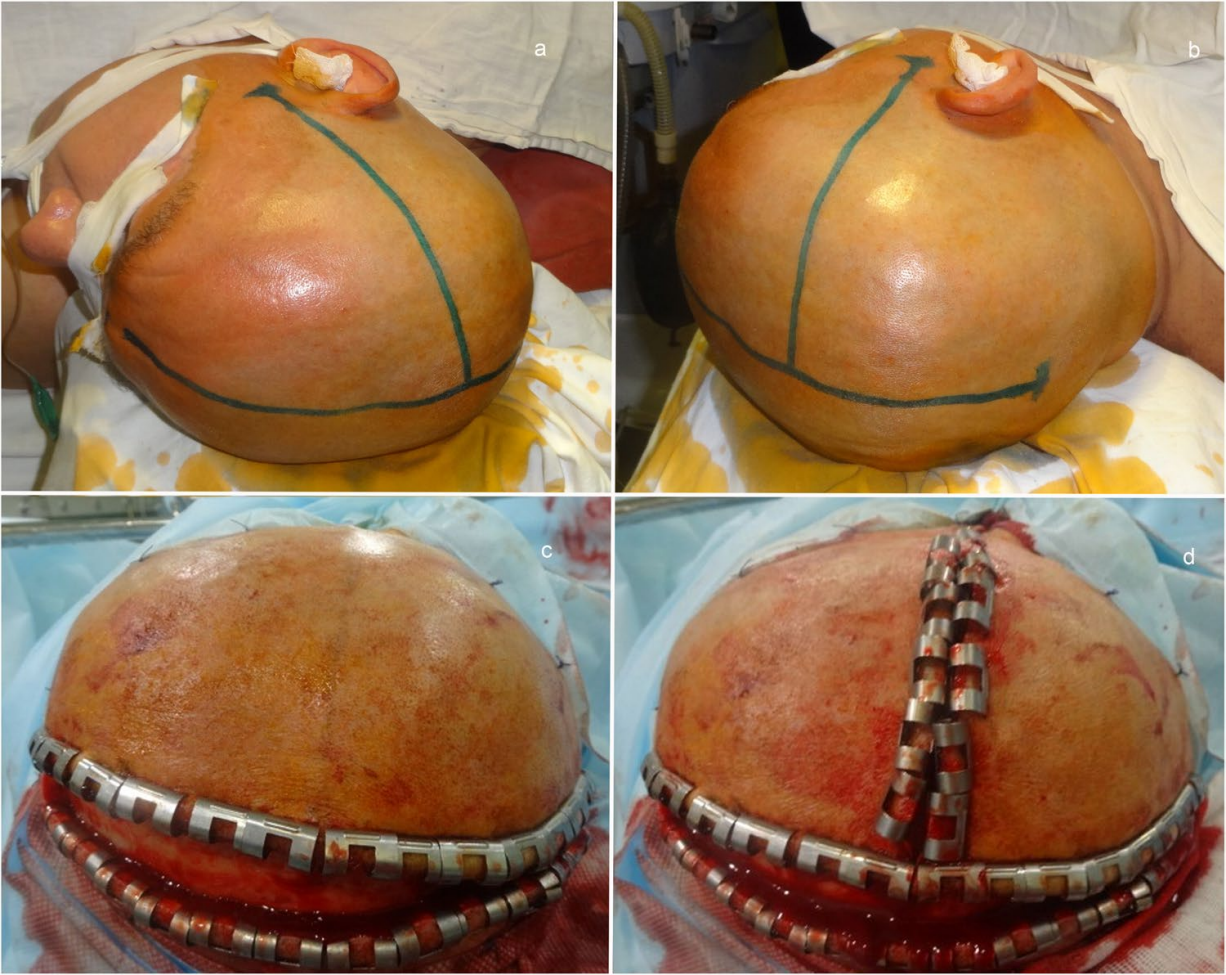
**a**, **b** Head mark for performance of a T-shaped skin incision. **c, d** Performance of a T-shaped skin incision. **c** First-stage skin incision along the sagittal suture line. **d** Second-stage vertical skin incision

General anesthesia is administered to the supine patient; after which, the neck and head are slightly retracted down to approximately 10°; then, the head is turned to the side opposite to the affected hemisphere so that the sagittal suture is almost parallel to the floor, and the zygomatic arch is the highest point of the surgical field. The shoulder on the affected hemisphere side is supported by means of a roll approximately 15 cm thick. Such position of the patient allows adequate and optimal access to the cranial base. Moreover, the patient’s shoulder does not limit the operating corridor, especially when the patient has a short neck or a large build. Marks are made for a T-shaped incision of the head skin. The first mark is made along the median line of the sagittal suture. Its anterior boundary is the head hairline, and it is continued posteriorly to the level of the external occipital protuberance. The second line is perpendicular to the first one and starts at 1 cm anterior to the tragus at the level of the zygomatic arch and then continues superiorly to cross the sagittal plane approximately by 4–6 cm posterior to the bregma (Fig. 1).

The skin and soft tissues are then incised. The first incision is made along the sagittal suture line, and then, the vertical incision is made starting from the zygomatic arch level and rising to cross the sagittal plane. The vertical incision is performed posterior to the superficial temporal artery trunk found by palpation (Fig. 1). Such skin incision preserves the blood supply of the anterior and posterior flaps from two territories, in contrast to the traditional question mark incision that may lead to development of necrosis of the skin flap.

**Fig. 2:**
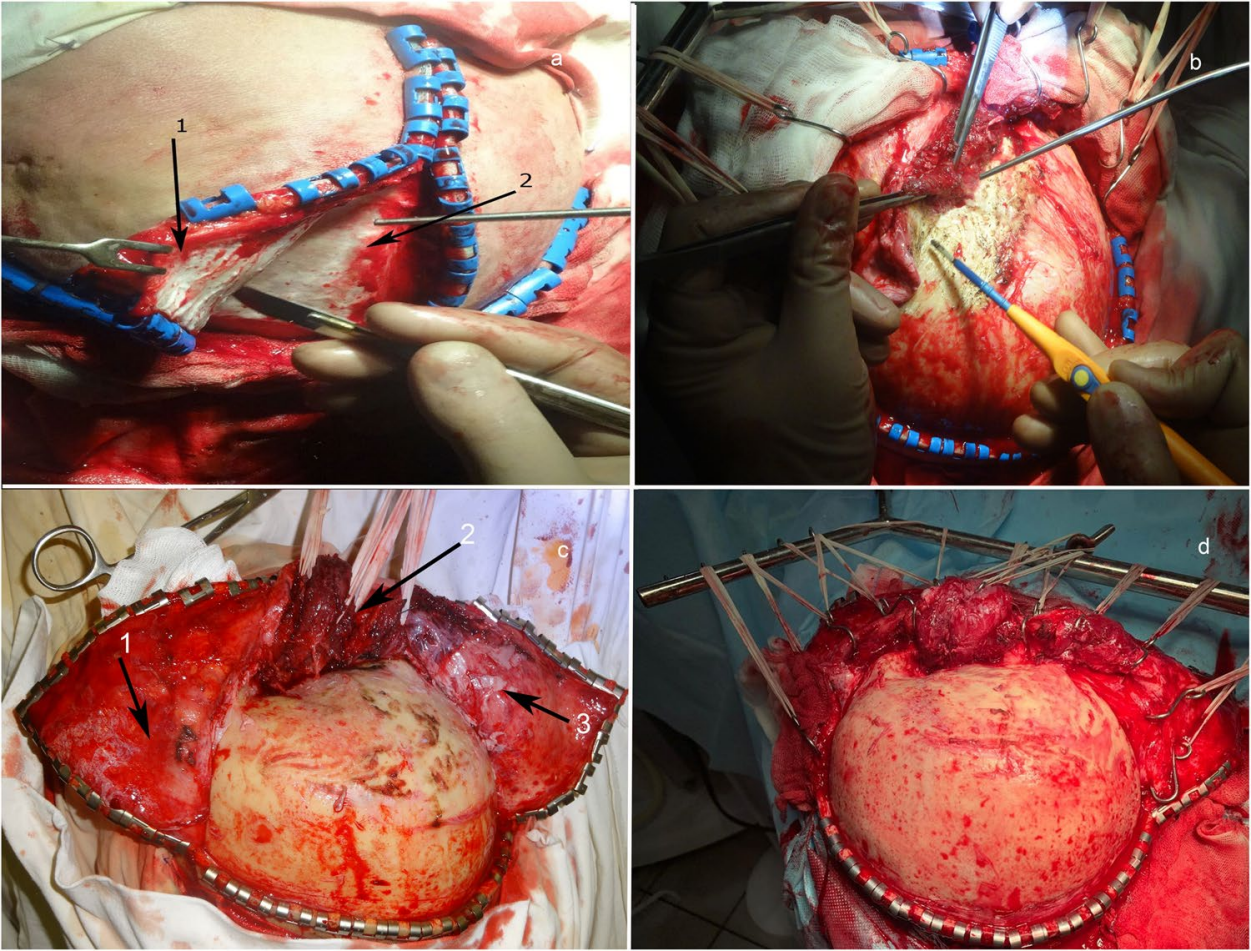
**a** Removal of a free skin flap by separation of the aponeurotic skin flap from the pericranium and the temporal muscle fascia. 1: aponeurotic skin flap; 2: pericranium. **b** Separation of the skin flap with the use of a monopolar coagulator. **c** General view after separation of the aponeurotic skin flap and the muscle flap with formation of the anterior and the posterior flaps. 1: anterior aponeurotic skin flap; 2: temporal muscle; 3: posterior aponeurotic skin flap. **d** Retractor for spreading of the surgical wound; ensures maximum retraction of aponeurotic skin flaps to the cranial base

Further, a free skin flap is obtained by incision and separation of an aponeurotic skin flap from the pericranium and the temporal muscle fascia (Fig. 2).

After visualization of the subcutaneous fat, incision is made of the temporal fascia along the entire length of the muscle approximately at 2 cm above and parallel to the zygomatic arch level to separate the aponeurotic skin flap together with the temporal muscle fascia, exposing its anterior third. The anterior aponeurotic skin flap together with the temporal muscle fascia is folded to the forehead to prevent it hanging over and interfering with the access to the cranial base. The anterior flap is separated to expose the frontozygomatic process and the anterior third of the zygomatic arch.

The posterior aponeurotic skin flap is separated to the level of the zygomatic arch and folded to the occiput. The pericranium is incised and separated above the level of the superior temporal line and folded over to the forehead. The temporal muscle is incised along the anterior and posterior boundaries in parallel to the muscle fibers and along the superior boundary in parallel to the superior temporal line, using a raspatory or a monopolar coagulator for separation (Fig. 2). The skin flap together with the fascia is folded away posterior and as far as possible inferior to the cranial base (Fig. 2), which, in its turn, allows exposure of not only the temporal squama but also the greater wing of the sphenoid bone to the base of the middle cranial fossa.

The formed aponeurotic skin flaps are folded as much as possible over to the cranial base with the aid of hooks. For this purpose, we spread the wound with a specially designed Y-shaped retractor (Fig. 2). This retractor ensures that retraction of both flaps to the cranial base is performed as atraumatically as possible.

### Craniotomy

**Fig. 3:**
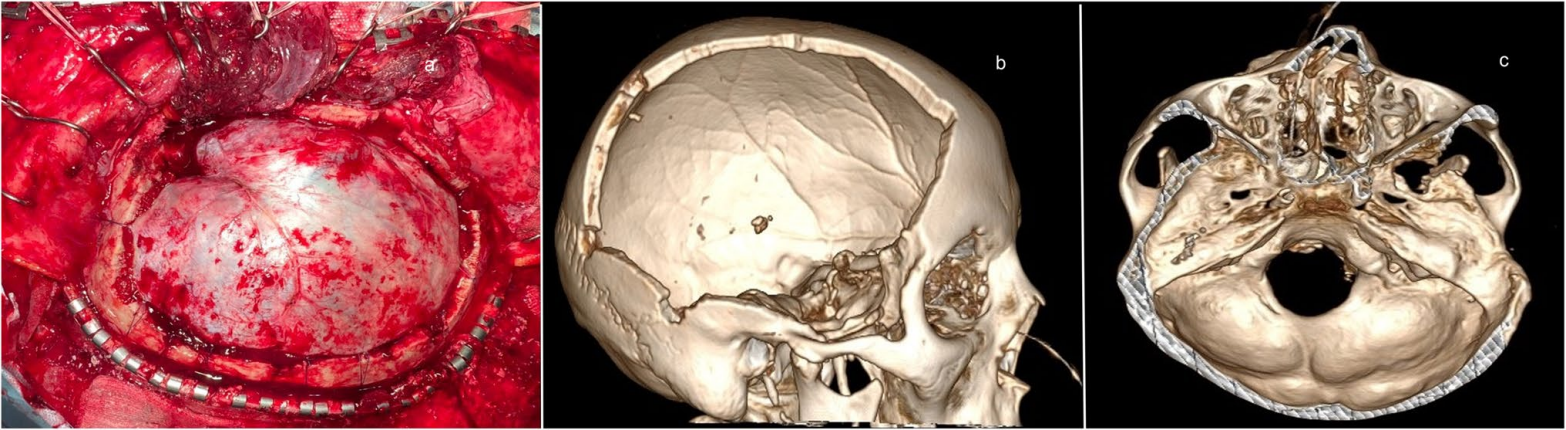
General right-side view of hemicraniectomy. **a** Intraoperative photo. **b, c** 3D MSCT images of the patient’s skull after performance of hemicraniectomy

An extensive bone flap with a size of not less than 12 × 14 cm is cut in the fronto-temporo-parieto-occipital zone. It is advisable to use multiple burr holes for cutting of the bone flap, to prevent damaging the dura mater, since it often adheres to the bone. The bone flap has the following boundaries: the upper boundary is at a distance of 2–3 cm laterally to the superior sagittal suture, to prevent hemorrhage from the arachnoid granulations. The anterior boundary is at the level of the frontozygomatic process. The posterior boundary extends to the level of the lambdoid suture. The inferior boundary in the posterior parts is at the level of the zygomatic process, above the asterion level, in order to prevent damaging the sigmoid and the transverse sinuses. In the anterior parts, resection of the temporal squama and of the greater wing of the sphenoid bone is performed to the level of entrance of the middle meningeal artery to the cranial cavity through the spinous foramen, which, in its turn, allows resection of the upright margin of the sphenoid wing (Fig. 3). Hemorrhage from the bone is controlled by means of medical bone wax. A fragment of collagen hemostatic sponge up to 1 cm wide is placed between the dura mater (DM) and the bone. Interrupted suturing is used to fix the DM to the aponeurosis in order to prevent epidural hemorrhage.

### Opening of the dura mater

**Fig. 4:**
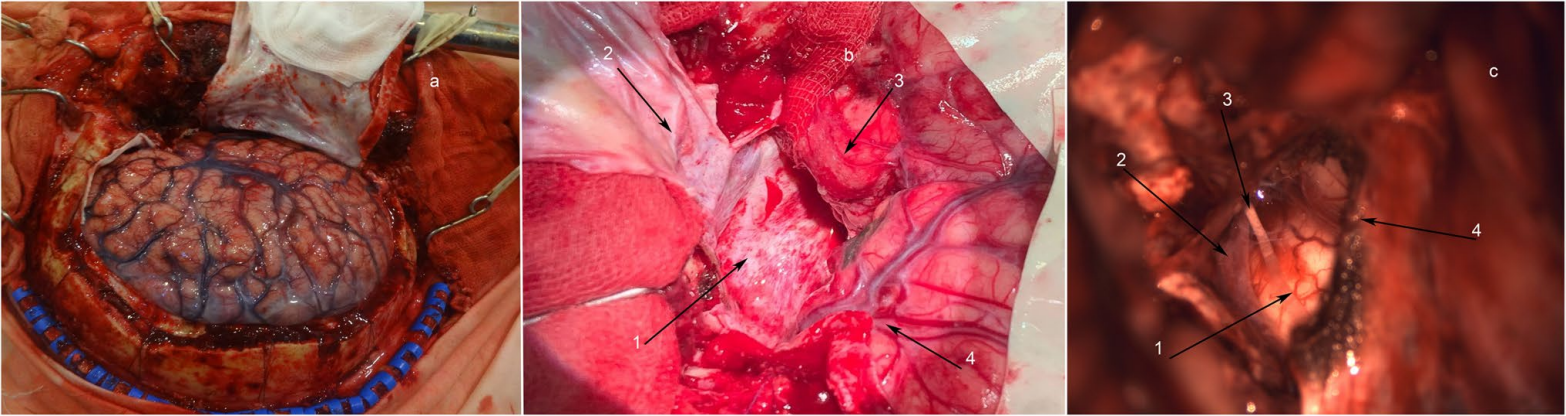
**a** General view of the surgical wound after hemicraniectomy and C-shaped incision of the DM. **b** General view after resection of the temporal pole. 1: floor of the middle cranial fossa after resection of the temporal pole; 2: DM folded over after opening; 3: temporal lobe (middle part); 4: frontal lobe. **c** General view after tentoriotomy. 1: pons; 2: right posterior cerebral artery; 3: right trochlear nerve; 4: cerebellar tentorium

The DM is opened by a C-shaped incision reflected toward the cranial base (Fig. 4).

Next, encephalotomy is performed, at a distance of 4 cm posterior to the temporal pole, perpendicular to the temporal gyri, from the superior gyrus level to the inferior gyrus; resection of the temporal pole is made by subpial aspiration to the level of the anterior boundary of the temporal horn, without amygdalohippocampectomy (Fig. 4).

The temporal lobe is retracted medially, posterior and inferior to visualization of the superior petrosal sinus and the cerebellar tentorium. Tentoriotomy is performed at a distance of up to 1 cm posterior to the superior petrosal sinus, including the free edge of the cerebellar tentorium (Fig. 4).

**Fig. 5:**
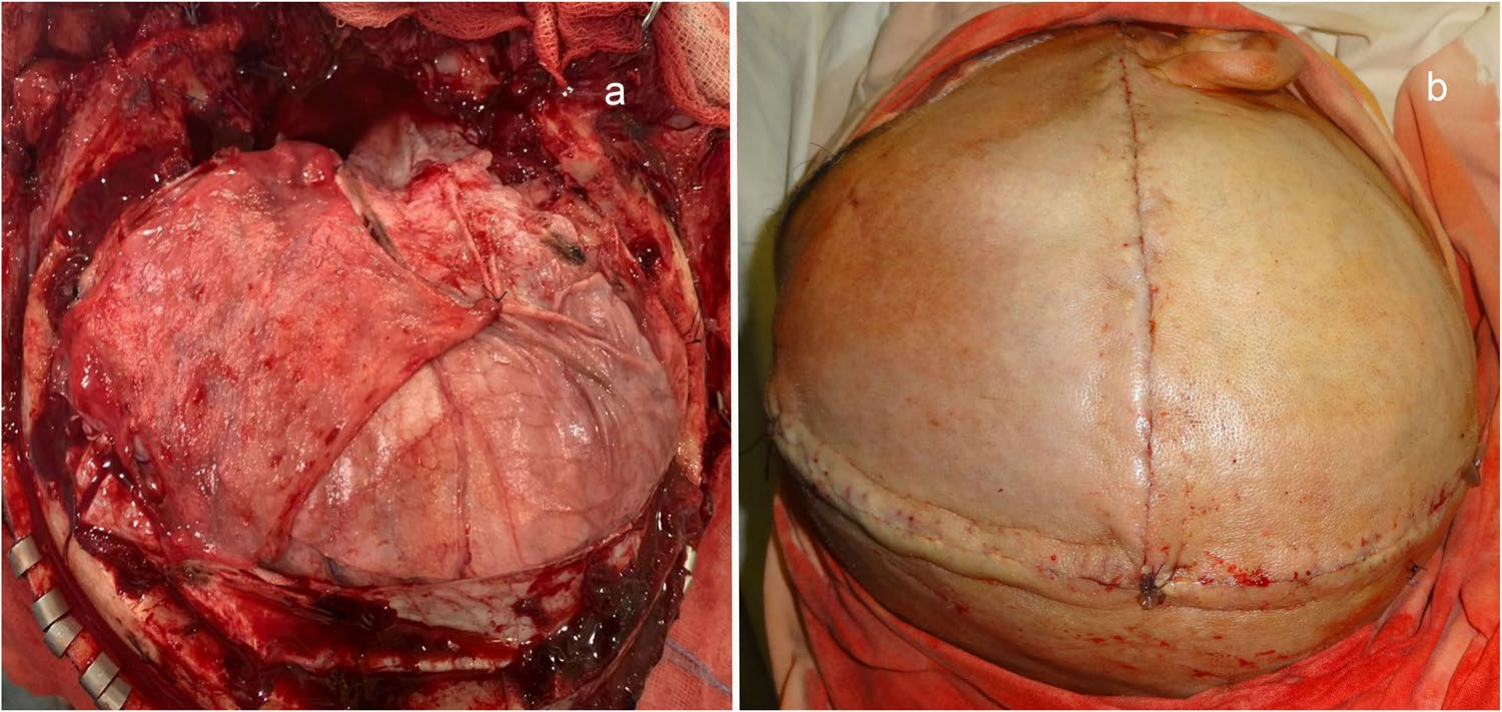
**a** DM defect plasty with the aponeurosis. **b** General view after layered closure of the surgical wound

DM plasty is performed using either artificial DM or a fragment of the aponeurosis, fixing the aponeurosis with interrupted sutures at multiple sites to prevent additional compression or shift of the brain. The bone flap is removed. Thorough hemostasis and layered closure of the wound are performed. The temporal muscle is sutured first and fixed to the aponeurotic skin flap, and then, we proceed by suturing of the vertical incision and then the sagittal incision; the skin is closed with a cosmetic subcuticular suture to prevent liquorrhea (Fig. 5). This surgical technique has been patented.

## Indications

Hemicraniectomy is indicated for malignant ischemic infarction in the territory supplied by the MCA. Besides, this method can be used for patients presenting refractory intracranial hypertension or transtentorial herniation in cases of intracerebral bleeding.

## Limitations

No limitations exist for the performance of this technique, which can always be used for malignant ischemic infarction in the MCA territory.

## How to avoid complications

Separation of the aponeurotic skin flap together with the temporal muscle fascia prevents damaging the facial nerve.

Resection of the temporal squama and of the greater wing of the sphenoid to the level of the middle meningeal artery allows resection of the upright margin of the middle cranial fossa, which ensures maximum decompression of the temporal lobe.

Resection of the anterior temporal lobe is the key supplementary factor of reduction of the intracranial hypertension and prevention of the development of transtentorial herniation.

Tentoriotomy is the main factor of decompression of the brain stem, especially after the development of transtentorial herniation.

Extensive tentoriotomy is not an option, because of the danger of brain matter herniation through the incision made.

## Specific perioperative consideration

It is imperative to determine the absence or presence of transtentorial herniation in patients with malignant ischemic infarction in the MCA territory, based on the results of multislice computed tomography (MSCT) of the brain.

Early rehabilitation and symptomatic therapy are performed during the postoperative period.

Mannitol is contraindicated during the postoperative period.

Stitches can be removed at day 12–14 postoperatively.

## Specific information to give the patient about surgery and potential risk

The patient and relatives must be informed about the operation scope, including resection of the extensive bone flap. The patient and relatives must be told that the operation will not stop the ischemic cascade and is life-saving. During the postoperative period, it is expedient to perform the bone defect plasty after stabilization of the patient’s condition.

The postoperative period course is severe and involves development of infection and inflammation complications.

## Supplementary Information

Below is the link to the electronic supplementary material.

Supplementary file1 (MP4 324037 KB)
